# Reinforcement learning increases wind farm power production by enabling closed-loop collaborative control

**DOI:** 10.1038/s44172-026-00667-8

**Published:** 2026-05-05

**Authors:** Andrew Mole, Max Weissenbacher, Georgios Rigas, Sylvain Laizet

**Affiliations:** Department of Aeronautics, Imperial College London, London, UK

**Keywords:** Wind energy, Computational science

## Abstract

Traditional wind farm control operates each turbine independently to maximize individual power output. However, coordinated wake steering across the entire farm can substantially increase the combined wind farm energy production. Although dynamic closed-loop control has proven effective in flow control applications, wind farm optimization has relied primarily on static, low-fidelity simulators that do not resolve critical dynamic turbulent fluctuations in the flow. In this work, we present a reinforcement learning controller trained using high-fidelity turbulence resolving simulations, enabling real-time response to atmospheric turbulence through collaborative, dynamic control strategies. In a three wind turbine test case, our reinforcement learning controller achieves a 4.30% (95% CI = [4.10%, 4.49%]) increase in wind farm power output compared to baseline operation, nearly doubling the 2.19% (95% CI = [1.98%, 2.39%]) gain from static optimal yaw control and a substantial increase over the gain from global wind direction based dynamic control obtained through Bayesian optimization of 2.67% (95% CI = [2.47%, 2.87%]). These results establish that reinforcement learning is able to utilize the increased information available from turbulence resolved simulations to learn improved, dynamic flow-responsive control for wind farm power maximization, with direct implications for accelerating renewable energy deployment to net-zero targets.

## Abstract (web summary)

Coordinated wind farm control is usually designed using simplified models. Andrew Mole and colleagues report a reinforcement learning controller, trained in turbulence resolving simulations, that responds dynamically to increase power output.

## Introduction

Wind energy is expected to play a crucial role in expanding renewable energy generation and decarbonizing energy production^1^. Maximizing the energy output of existing wind farms through wind farm control is a key strategy to enhance the efficiency of renewable energy systems. Modern large-scale offshore wind farms consist of multiple turbines grouped together, usually in well-structured formations. The wind turbines are typically operated with a greedy strategy, optimizing their individual power generation whilst ignoring the effect on other wind turbines. This leads to individual wind turbines often operating in suboptimal conditions due to aerodynamic interactions between the wind turbines, resulting in a reduction of the total power output of the wind farm^2^. By instead implementing a collective wind farm control approach, that accounts for aerodynamic interactions between turbines, wake effects can be mitigated, leading to improved wind farm efficiency^3,4^.

Efficient control of wind farms remains one of the major challenges in wind energy science^5^ and has been the focus of extensive research. Control strategies fall broadly into two categories: *static* (or *quasi-static*), where fixed optimal parameters are obtained for given conditions, and *dynamic*, where parameters change as a function of time. Dynamic wind farm control can be further divided into *open-loop control*, where control strategies follow a predetermined dynamic behavior, and *closed-loop control*, where the controller senses and actively responds to changing flow conditions. Various models are used to simulate wind farms, ranging from lower-fidelity wake models^6–8^ to higher-fidelity large eddy simulations (LES) solvers^9,10^. Analytical wake models are computationally efficient but sacrifice accuracy, particularly in capturing transient flow dynamics and wake-to-wake interactions. The most commonly studied control methods for wind farm optimization are axial induction control and yaw control, with yaw control generally showing more promising improvements in power generation^11^. Numerous studies have focused on identifying static optimal settings for these actuations, using a variety of optimization techniques^3,12,13^, including surrogate-based Bayesian optimization. Open-loop dynamic control has been studied by applying periodic forcing for induction control^14,15^, and to promote wake mixing through helical pitch control^16^. Although these methods explore promising mechanisms for collaborative control, the prescription of a predetermined temporal actuation means that they do not adapt to evolving wind conditions, which limits their effectiveness in realistic environments.

Several approaches have implemented closed-loop wind farm control through model-predictive control (MPC) using dynamic wake models combined with state estimation methods^17,18^. The use of a reduced-order model in this framework means that instantaneous turbulent structures can not be accounted for. Closed-loop control using high-fidelity flow information has also been investigated. Notably, Munters and Meyers^15^ used adjoint-based optimization in LES to compute dynamically optimal yaw and induction actuation. While these LES-based controllers achieve performance improvements, their computational cost makes them unfeasible for practical control. We refer the reader to the review by Meyers et al.^11^ for an in-depth review of wind farm control studies.

Data-driven methods have emerged as a competitive alternative to classical control frameworks due to their ability to adapt to complex system dynamics as data become available. Reinforcement Learning (RL) is a machine learning paradigm in which a controller (or agent) learns to make sequential decisions by interacting with an environment and receiving rewards based on its actions^19^. Well known for achieving superhuman performance in video games^20,21^, RL has since been successfully applied to flow control applications such as the reduction of drag in a 3D turbulent flow^22^ and the suppression of vortex shedding in partially observable environments^23^. We highlight the recent work by Font et al.^24^, who couple an RL control framework with a high-fidelity 3D flow simulation of a turbulent separation bubble. They demonstrate superior performance of RL compared to classical open-loop periodic forcing control. We refer the reader to the recent review by Vignon et al.^25^ for an overview of flow control with RL.

RL has been explored for the optimization of wind farms, but most studies have been limited to static control policies. The majority of existing work^26–30^ have trained RL controllers using analytical wake models as the environment which capture only the steady-state, time-averaged flow field. These models neglect the transient dynamics of the atmospheric boundary layer and wake interactions, making it impossible to learn dynamic closed-loop controllers. More limited work has explored training RL using dynamic wake models which introduce simplified unsteady turbine and wake dynamics^31,32^. However, these models still rely on reduced order physics and cannot fully represent the three-dimensional turbulence, wake meandering, and turbine wake interaction processes, especially when effected by dynamic turbine actuation. As a result, the literature lacks closed-loop control strategies that are trained using full-field, turbulence-resolving flow information, leaving open the question of how controllers might perform when given access to information of the unsteady turbulent wind conditions. Korb et al.^33^ couple a PPO^34^ RL controller with a 3D LES simulation of three in-line turbines, controlling both generator torque and fixed-frequency blade pitch oscillations. However, neither approach results in an effective dynamic closed-loop strategy with the torque control failing to increase power output, while pitch control converges to a nearly static amplitude, effectively converging to an open-loop forcing controller. To date, no studies have successfully demonstrated an RL controller that achieves efficient, dynamic, closed-loop wake steering based on real-time flow observations in a high-fidelity simulation environment.

In the present work, we address this gap by combining an RL control framework with high-fidelity LES of a small three-turbine wind farm. We obtain a closed-loop controller that dynamically adapts the turbine yaw angles in response to the turbulence in the atmospheric boundary layer to control the wind conditions around the farm in a way that increases the wind farm power output. The contribution of this work lies not in the use of yaw control or reinforcement learning, but in demonstrating that access to turbulence-resolved flow information enables qualitatively different, flow-responsive control strategies that outperform static and quasi-static baselines in a high-fidelity setting.

## Results

### RL training and Bayesian optimization

**Fig. 1:**
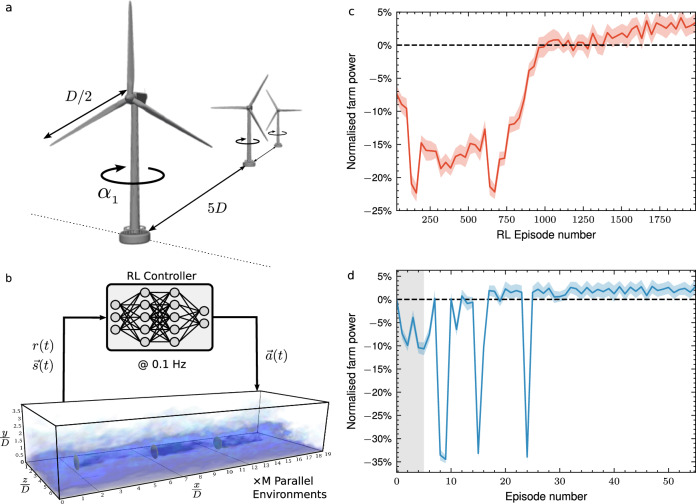
Wind farm and reinforcement learning (RL) setup and training. **a** Schematic of the simulated wind farm. Three in-line turbines with diameter *D* = 126 m in an atmospheric boundary layer are simulated using a high-fidelity 3D-LES solver. The turbines are aligned along the direction of the flow and the yaw angle is dynamically adjusted. Turbine geometry adapted and modified from “NREL 3 Blades 5MW Rotor” by Eduardo Firvida (Pinshape), licensed under Creative Commons Attribution (CC BY 4.0). **b** Schematic of the RL control loop. The controller receives velocity sensor observations from *M* = 32 large eddy simulation environments and determines the yaw angles of three in-line turbines. Angles are updated by the controller every 10*s* of simulated time. **c** Mean farm power during RL training relative to the greedy baseline with 95% CI computed over *n* = 32 training environments as a function of episode number. **d** Mean relative farm power with 95% CI computed over *n* = 32 training environments during Bayesian optimization to find optimal static yaw angles.

A set of baselines is established in order to fairly assess the performance of the discovered RL control strategies. In standard wind farm layouts, individual turbines face directly into the average oncoming wind, which in our case of three in-line wind turbines (as in Fig. 1a) corresponds to ${\vec{\alpha }}_{{{\rm{greedy}}}}=({0}^{\circ },{0}^{\circ },{0}^{\circ })$. We refer to this as the ‘greedy baseline’. In addition, we use a Bayesian optimizer (see Methods section) to compute the optimum *static* angles of individual turbines. The optimization progress is shown in Fig. 1d, initially exhibiting substantial fluctuations with peaks below  − 30% farm power, reflecting the exploration as Bayesian Optimization (BO) samples broadly across the parameter space. As optimization progresses, the resulting wind farm power consistently exceeds the greedy baseline. The last 20 iterations of the BO exhibit minimal improvement with the farm power fluctuating within a narrow band. This plateauing is consistent with the fact that the BO has exhausted the exploitable regions of the search space. The optimal (static) turbine yaw angles found by the BO are ${\vec{\alpha }}_{{{\rm{BO}}}}=(2{0}^{\circ },1{3}^{\circ },-{3}^{\circ })$. These angles show a decrease from the upstream to the downstream turbines, which is consistent with established wake steering strategies^13^, where optimal power production is achieved when the upstream turbines are yawed the most, with reduced yaw angles for the downstream turbines.

We additionally include a *dynamic* BO baseline that approximates a quasi-static closed-loop strategy. In this case, BO is performed offline using an analytical wake model to determine the optimal turbine yaw angles for a discrete set of mean wind directions. The resulting optimization map (see Supplementary Fig. 1) forms a lookup table that the controller queries in the LES environment using an instantaneous upstream wind-direction sensor, allowing the yaw settings to adapt to changing inflow conditions.

In order to ensure effective RL convergence, we conduct a set of hyperparameter sweeps. We find that using a larger number of velocity sensors located in the vicinity of the wind farm is advantageous, and that carefully tuning the entropy learning rate (a hyperparameter that controls the degree of exploration by the agent as training progresses by encouraging randomness in its policy) is imperative to prevent premature convergence to a local optimum. We train our RL controller using the selected hyperparameters (see Supplementary Table 1) for a total of 1 × 10^6^ interactions collected across 32 parallel LES environments, see Fig. 1b. This number of training interactions corresponds to approximately 116 days of simulated wind-farm operation under constant wind conditions. The progress of the RL training is shown in Fig. 1c with the RL control initially reducing farm power output, including two pronounced troughs below  − 20%, as the agent explores the policy space driven by the regularization of the entropy of the objective. After approximately 1000 episodes, the policy consistently increases the wind farm power output over the greedy baseline, and continues to improve the performance gradually as it transitions from broad exploration to more focused exploitation of the policy. Training the RL controller in the simulated environments required 18.5 h on 34 dual AMD EPYC 7742 64-core processor nodes on the ARCHER2 system^35^. Energy consumption was recorded using ARCHER2’s job-level energy accounting tools and totaled approximately 254 kWh per training run.

### Mean farm power increase

We evaluated the RL controller, the static BO, the dynamic BO, and the greedy baseline by collecting a total of *n* = 224 episodes across 16 parallel LES environments with each using an independent turbulent realization of the same neutral atmospheric boundary-layer conditions at the inlet. Each episode lasts 2 × 10^4^*s* and the power signal is sampled at 5 Hz. The mean farm power $\bar{P}$ is calculated by averaging the instantaneous power over the duration of an episode.

**Fig. 2:**
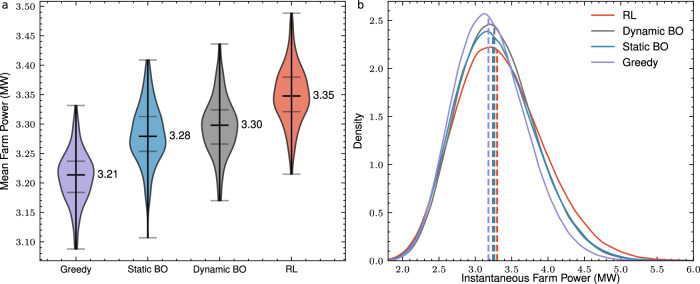
Power distribution comparison between reinforcement learning and other control strategies. **a** Violin plot of the mean farm power in MW computed over *n* = 224 evaluation episodes across 16 environments. The horizontal lines are (bottom to top): minimum, first quantile, median, third quantile, maximum. The annotated value is the median. **b** Density of the *instantaneous* power output normalized so that the integral of each curve is the mean farm power. The dashed vertical lines indicate the medians of the distributions. The RL controller achieves a higher mean power by spending more time in higher power output conditions.

Figure 2a shows the distribution of the mean farm power for each case. Relative to the greedy baseline, the static Bayesian optimum increases the mean farm power output by 2.19% (95% confidence intervals (CI) = [1.98%, 2.39%]), and the dynamic BO by 2.67% (95% CI = [2.47%, 2.87%]), while the RL controller increases the mean power by 4.30% (95% CI = [4.10%, 4.49%]). The magnitude of the increase in power for each strategy depends on the conditions of the atmospheric boundary layer, as well as the specifications and layout of the wind turbine.

The RL controller robustly increases the mean farm power while outperforming both the greedy baseline and both static and dynamic BO controllers. The dynamic BO controller performs better than the original static BO baseline, suggesting that a quasi-static application of BO can improve the performance. However, the dynamic BO does not match the performance of the RL controller which consistently achieves higher power production. The higher mean power is achieved by spending more time occupying higher power generation states. This can be seen in the plot of the instantaneous power output distributions in Fig. 2b. The peak of the distribution is reduced relative to the greedy case, showing a more variable power output with a higher distribution across the high-power tail of the distribution. A similar, though less pronounced, shift is observed for the Bayesian optimization cases, in line with the more modest power increases.

### Closed-loop controller test

We verify that the trained RL controller is performing active *closed-looped* control as opposed to having learned an open-loop forcing strategy. To accomplish this, we evaluate the agent on 16 environments and save the sequence of actions performed. We then “replay” this sequence of actions on 16 environments with *statistically independent* inlet conditions. We collect one episode containing 2 × 10^4^*s* per environment (*n* = 16 samples in total) and compute the mean power $\bar{P}$ for each. In this test, the “replayed” controller does not result in a statistically significant increase in power (95% CI = [ − 0.35%, + 0.96%]) compared to the greedy case. We conclude that, because accurate sensor measurements are required for the RL controller to find the power improvements, the learned RL controller is closed-loop; it actively responds to the changing flow conditions instead of forcing the flow at a certain fixed frequency.

### Dynamic switching

**Fig. 3:**
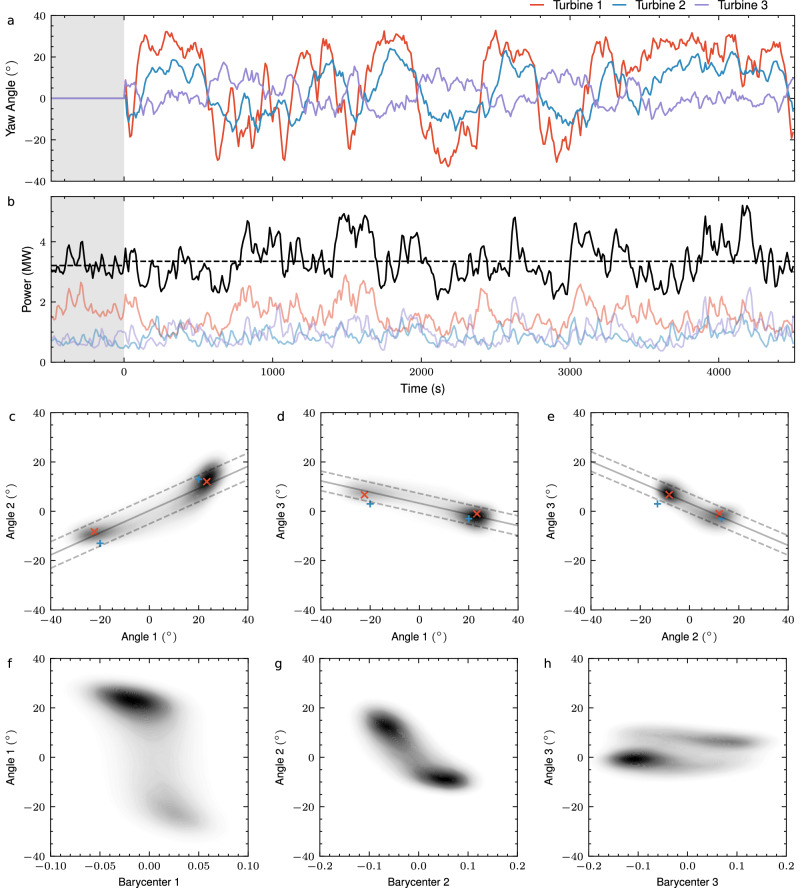
Reinforcement learning (RL) produces coordinated and flow-responsive yaw control. **a** Turbine yaw angles and **b** power output for an evaluation episode of the converged RL controller. The controller starts at time *t* = 0*s*, and the mean power (dotted horizontal black line) increases. The black line in the power plot is the total wind farm power (the sum of the individual turbine powers). **c**–**e** Joint densities of angle pairs after correcting for time shifts. Time shifts are found using cross-correlation analysis. Linear models are fitted using least squares error. Red crosses show the peaks of the RL densities ${\vec{\alpha }}_{{{\rm{RL}}}}$ and the blue plus signs show the Bayesian static optimum locations and their symmetric inflections ${\vec{\alpha }}_{{{\rm{BO}}}}$. **f**–**h** Joint densities between the angle of each turbine and the barycenter of the velocity in front of that turbine.

Figure 3a, b show a time series segment of an evaluation rollout of the RL controller, showing the yaw angles and corresponding power outputs. Initially, all yaw angles are at 0^∘^, corresponding to the pre-control baseline. When the controller is activated, the turbines’ yaw angles are dynamically adjusted, following the learned policy, with a combination of oscillations and discrete switching between yaw states. The resulting power output signals show strong fluctuations, driven in part by variability in the atmospheric boundary layer. The mean power generation improvement exhibited by the RL controller is obscured by these fluctuations. Consistently with established wake steering strategies, the controller reduces the power generation of the first turbine, which is offset by the increased energy capture of the downstream turbines. Although the time series illustrates the dynamic behavior of the RL controller during a single rollout, to better understand its behavior, statistical characterizations are drawn from the *n* = 224 evaluation episodes. These distributions reveal consistent control patterns and performance trends that are not apparent from individual trajectories alone.

Firstly, a cross-correlation analysis of the turbine yaw angles (see more details in Supplementary Fig. 2) is conducted, showing distinct peaks at specific time delays. Relative to the angle of the first turbine, the angle of the second turbine is best correlated with a delay of  + 70 s. This is approximately the advection time based on the free-stream flow (*U*_0_ = 7.5 ms^−1^) from one turbine to the next (*Δt* = 5*D*/*U*_*∞*_ = 84 s). The RL controller has therefore learned that there is an inherent time delay in the system, created by the finite speed of propagation of the fluid flow. Interestingly, the third turbine correlation peak has a delay of  − 30 s relative to the second, meaning that the third turbine acts before the second, a fact the authors attribute to the fact that the propagation time between these turbines is not an important feature for power extraction and the third turbine has no downstream turbines to account for.

After compensating for time lags, the turbines appear to move in synchronization. Figure 3c–e shows the joint densities of the yaw angle signals when adjusting for the time delays, revealing a strong positive correlation between the first and second turbines and a negative correlation between the rear turbine and the others. This hypothesis is verified by fitting linear models to the yaw angle pairs. Good agreement is found, with *R*^2^ = 0.7114 for the front pair (*α*_2_ vs. *α*_1_), and *R*^2^ = 0.5370 and *R*^2^ = 0.5358 for the rear turbine’s relationships to *α*_1_ and *α*_2_, respectively and all mean residuals below 4. 3^∘^. The details of the fitted linear models are given in the Supplementary Information.

Closer inspection of the density plot in Fig. 3 reveals that the yaw angles tend to concentrate around two local modes ${\vec{\alpha }}_{{{\rm{RL}}}}^{+}=(2{3}^{\circ },1{2}^{\circ },-{1}^{\circ })$ and ${\vec{\alpha }}_{{{\rm{RL}}}}^{-}=(-2{2}^{\circ },-{8}^{\circ },{7}^{\circ })$ (rounded to nearest integer). The modes are close in absolute value to the Bayesian static optimum ${\vec{\alpha }}_{{{\rm{BO}}}}=(2{0}^{\circ },1{3}^{\circ },-{3}^{\circ })$ and its symmetric inflection $-{\vec{\alpha }}_{{{\rm{BO}}}}$, respectively. Furthermore, when using ${\vec{\alpha }}_{{{\rm{RL}}}}^{\pm }$ as static angles, the mean power generated is nearly identical to the power generated by the static optimum ${\vec{\alpha }}_{{{\rm{BO}}}}$: When used as static angle configurations, ${\vec{\alpha }}_{{{\rm{RL}}}}^{+}$ generates  − 1.36% and ${\vec{\alpha }}_{{{\rm{RL}}}}^{-}$ generates  − 1.59% less power than the optimal static angle ${\vec{\alpha }}_{{{\rm{BO}}}}$. Since the mean flow through the turbines is statistically symmetric in the stream-wise direction, it is expected that $\vec{\alpha }$ and $-\vec{\alpha }$ generate the same power. However, this symmetry holds only in a long-time average sense, with the instantaneous flow field being highly asymmetric due to unsteady phenomena, such as turbulent gusts, breaking this symmetry at any given moment.

We hypothesize that the RL controller has learned to exploit the instantaneous asymmetries by dynamically switching between the two symmetric static optima ${\vec{\alpha }}_{{{\rm{BO}}}}$ and $-{\vec{\alpha }}_{{{\rm{BO}}}}$. In fact, the strategy switches between ${\vec{\alpha }}_{{{\rm{RL}}}}^{+}$ and ${\vec{\alpha }}_{{{\rm{RL}}}}^{-}$, which when used as static angles produce a mean power very close to that of the Bayesian optima.

Further insight into the controller’s sensing strategy is provided by examining the joint density plots between the yaw angle of each turbine and the lateral position of the velocity barycenter directly upstream (Fig. 3, bottom row). For each turbine *t*, the barycenter ${\left\langle z\right\rangle }_{t}$ is computed from the stream-wise velocity measured at fourteen probes located 1*D* and 1.5*D* upstream of each turbine at hub height. This is a subset of the signals used as observations by the RL controller. The discrete barycenter is given by:

1 $${\left\langle z\right\rangle }_{t}=\frac{{\sum }_{i=1}^{14}{u}_{i}(z-{z}_{t})}{D{\sum }_{i=1}^{14}{u}_{i}},$$

where *u*_*i*_ is the stream-wise velocity measured at lateral position *z*_*i*_. For the first and second turbines, a clear negative correlation is observed between the barycenter and the yaw angle (see Fig. 3f–h) meaning that when the incoming flow shifts laterally in one direction, the RL controller responds by yawing the turbine to deflect the flow in the opposite direction. This indicates that the controller exhibits a reactive steering mechanism for which the turbines counteract the incoming asymmetry to redirect higher-momentum flow toward the downstream turbines. For the third turbine, the correlation is less clear, possibly because the incoming flow is more intermittent as a result of wake-to-wake interactions. The control of the third turbine does not need to balance its power output with its effect on the other turbines (as there are no turbines downstream of it), which explains its different response to the flow fluctuations in order to maximize its own power extraction. Overall, these correlations suggest that the RL controller has not only learned time-coordinated behaviors but also developed a spatially aware policy that maps upstream flow features to turbine actuation in a responsive, feedback-driven manner.

### Spectral densities and Bandwidth-limiting analysis

**Fig. 4:**
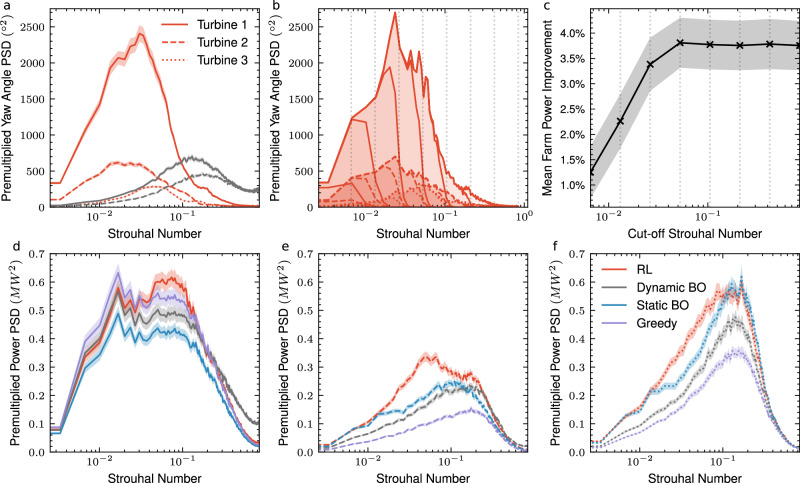
Spectral characteristics of yaw control and their impact on wind farm power. **a** Premultiplied power spectral density of turbine yaw angles of the controller. **b** Power spectral density of turbine yaw angles with various low-pass filters applied shown by dotted vertical lines and progressively darker shading indicates stronger low-pass filtering. **c** The corresponding mean farm power improvements for the different low-pass filtered controllers. **d**–**f** Premultiplied power spectral density of the individual turbine powers (turbine 1, 2, 3 respectively). Colors are consistent across all panels: uncontrolled (greedy) case (purple), static Bayesian optimum (BO) (blue), dynamic BO (gray), and dynamic reinforcement learning control (red). Shaded areas show the 95% CI computed over *n* = 224 evaluation environments.

A spectral analysis of the yaw angle signals obtained from the RL evaluation run reveals that the dominant frequency component is located at St ≈ 2 × 10^−2^, see Fig. 4a. Here, the Strouhal number St = *fD*/*U*_0_, with *D* the turbine diameter, *U*_0_ the free-stream velocity at turbine hub height and *f* the measured frequency. The yaw angle is actuated across a broad range of frequencies, suggesting that it is targeting turbulent features across a wide range of temporal scales. The dominant actuation frequency is far from the wake meandering (St ≈ 2 × 10^−1^) and vortex shedding (St ≈ 3 × 10^−1^) frequencies and is also at a lower frequency than the dominant frequency in the dynamic BO evaluation. The resulting frequency response of the three turbines individual power output for each of the greedy, static BO, dynamic BO and RL cases are shown in Fig. 4(d–f). Compared to the both the BO cases, which reduce the strength of the power fluctuations of the first turbine compared to the greedy case, the RL case only marginally reduces the power fluctuations at low frequencies and marginally increases them in the range 0.05≤*St*≤0.1. The dynamic BO raises the PSD at the highest frequencies for the first turbine, a behavior which is not present in the RL case. The second and third turbines see greater increases in power fluctuation in the RL case than in the BO cases. This is likely due to the increase in the wind speeds and subsequent mean power output of these turbines.

To assess the relative importance of each frequency range of the RL actuation, we conducted a bandwidth-limiting study. We use Welch’s method to low-pass filter the yaw angle signal from an evaluation run at decreasing cut-off frequencies starting at half the Nyquist frequency; and then replay the filtered angle signal in the environments with inlet conditions *identical* to the conditions used to compute the original angle signals. The result is that we are able to limit the effective frequency from the original 0.1 Hz to 3.125 × 10^−3^ Hz, see Fig. 4c, d). The maximum angular yawing velocity experienced by the turbines is reduced by more than half from 1^∘^s^−1^ to below 0. 4^∘^s^−1^.

### Flow analysis

**Fig. 5:**
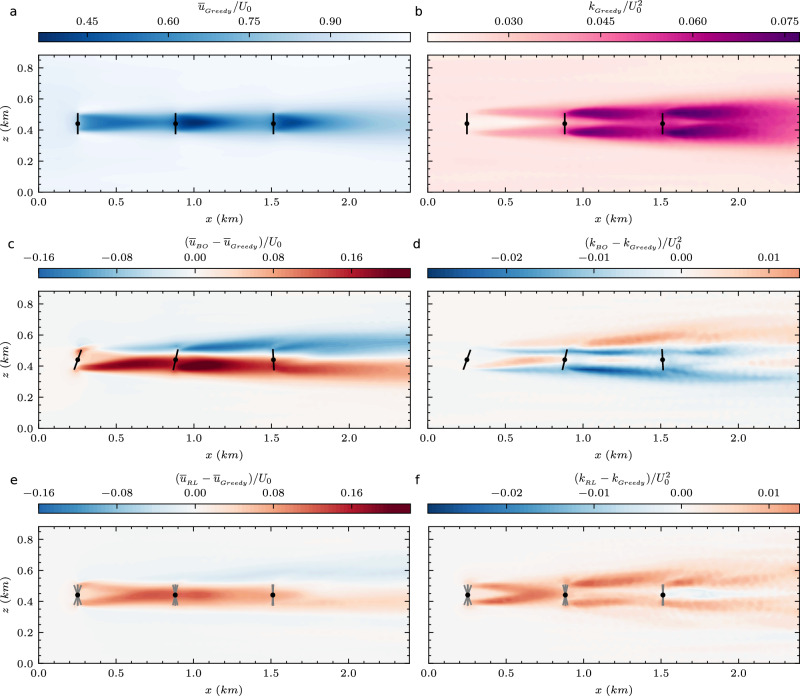
Flow field changes induced by static and reinforcement learning (RL) yaw control strategies. Slices of the flow field are taken at the wind turbine hub height showing **a** the time-averaged stream-wise velocity and **b** the resolved turbulent kinetic energy of the flow around the turbines in the greedy case. The change in the **c** time-averaged stream-wise velocity and **d** the resolved turbulent kinetic energy, when the turbines are yawed at constant angles found by the Bayesian optimization. The change in the **e** time-averaged stream-wise velocity and **f** the resolved turbulent kinetic energy, when the turbines are controlled actively by the RL controller.

To evaluate the effect of the control strategy on the flow field around the wind farm, we compare the time-averaged stream-wise velocity and resolved turbulent kinetic energy (TKE) fields on slices at the turbine hub height for the baseline greedy control, the static optimal configuration obtained via BO, and the RL controller (Fig. 5).

In the greedy case (Fig. 5a, b), the reduced velocity of the wind behind the turbines aligns with the downstream turbine locations, which is the cause of the reduced power output of the second and third turbines. The velocity gradients caused by the turbines result in an increase in the turbulent kinetic energy, particularly concentrated immediately behind the turbines.

When the turbines are statically yawed at the angles determined by the BO, the wakes are deflected laterally. This results in both a relocation in the position of the wake and a partial recovery in flow speed. The second and third turbines in this case experience an increase in mean velocity on one side and a decrease on the other relative to the greedy configuration (Fig. 5c). When averaged over each of these turbines swept area, the net effect is an increase in the wind speed and a corresponding increase in the power outputs of these turbines. The turbulent kinetic energy shows an overall reduction in (Fig. 5d), with localized increases near the edges of the deflected wakes.

In the RL-controlled case, the time-averaged velocity field around the three turbines shows reduced wake intensity and higher mean wind speeds compared to the baseline. Notably, the increase in the averaged wind speed is concentrated at the center-lines of the second and third turbines, where power recovery is most critical. Additionally, the RL controller induces a broad increase in the turbulent kinetic energy, in contrast to the redistribution observed with the static BO control. This increased turbulence within the wake may contribute to improved mixing and accelerated wake recovery, contributing to the overall increase in power output achieved by the RL strategy.

**Fig. 6:**
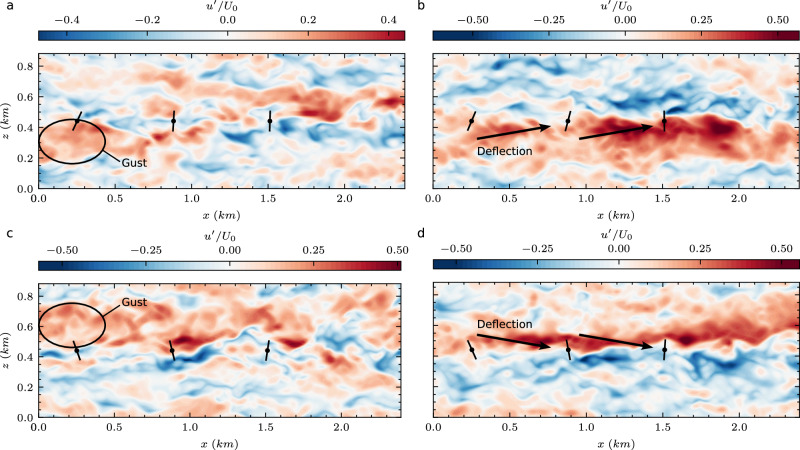
Reinforcement learning controller dynamically steers incoming gusts towards downstream turbines. Slices of the flow field at the wind turbine hub height showing the instantaneous fluctuations of the stream-wise velocity at different times in the simulation **a** *t* = 1250 s **b** *t* = 1500 s **c** *t* = 2000 s **d** *t* = 2250 s.

To further highlight the RL controller’s dynamic and adaptive behavior, we analyze instantaneous snapshots of the stream-wise velocity fluctuations ${u}^{{\prime} }=u-\overline{u}$, at hub height across four time instances (Fig. 6). At *t* = 1250 s (Fig. 6a), a high-speed gust originating from the atmospheric boundary layer approaches the first-row turbine from the right side of the wind farm. In response, the RL controller yaws the first turbine to deflect the gust toward the centerline, guiding the high-momentum flow toward the downstream turbines. By *t* = 1500 s (Fig. 6b), the second turbine has also adjusted its orientation to continue steering the gust towards the third turbine, maximizing its beneficial impact. The symmetric controller behavior is shown later in the simulation at *t* = 2000 s (Fig. 6c), a gust approaches from the right side, and again the upstream turbines yaw angles are adjusted to redirect the incoming higher momentum wind toward the center as seen at *t* = 2250 s (Fig. 6d). This behavior demonstrates the RL controller’s ability to sense asymmetries in the incoming flow and coordinate the turbine yaw angles in real time to opportunistically capture transient increases in available wind energy. The switching behavior between two dominant yawing modes, previously characterized as ${\vec{\alpha }}_{{{\rm{RL}}}}^{+}$ and ${\vec{\alpha }}_{{{\rm{RL}}}}^{-}$, is reflected here in the gust directionality and the controller’s selection of the corresponding coordinated yaw pattern. This dynamic switching, combined with flow-aware coordination, underlies the ability of the RL controller to outperform static BO-derived yaw configurations.

## Discussion

In the present study, we explored the potential of collaborative dynamic wake steering to optimize the power output of a wind farm. We coupled a high-fidelity LES of a small wind farm with an RL controller, allowing the controller to dynamically update the yaw angles of the turbines by sensing the fluctuations in the velocity field around the turbines. The converged RL controller yields a  + 4.3% increase in power over the greedy baseline, an improvement of almost a factor 2 over the increase in power obtained with static optimal angles found using Bayesian optimization.

The learned control strategy is analyzed in detail. First, we verified that the controller is indeed closed-loop, meaning that the strategy actively takes advantage of the sensor information. By analyzing the cross-correlation between turbines, we showed that the controller dynamically switches between two almost-symmetric local optima, which are close to the optima found using static Bayesian optimization. We confirm that this switching is based on the location of wind gusts formed in the atmospheric boundary layer. A detailed analysis of the flow fields shows how the implementation of the controller changes the wind conditions around the turbines allowing for the increased power capture.

The learned controller operates at an effective frequency of 3 × 10^−3^ Hz, with a maximum angular velocity of less than 0.4 deg s^−1^, which is likely achievable for latest-generation commercial wind turbines. However, implementing such controllers in operational wind farms involves challenges related to computational demands, sensing requirements, and real-world transferability, in addition to an analysis of component fatigue.

One barrier is the computational cost of training. Our RL agent requires thousands of episodes to converge, which is only feasible in simulation with parallelization of the environments. Collecting this experience solely through real-world online training is impractical due to the slow dynamics of wind farms and the associated operational risks. Consequently, deployment relies on training using high-fidelity simulated environments, with the trained policy then exported to run in real time.

Fortunately, once trained, the policy’s neural network is computationally inexpensive to evaluate and can produce control decisions in real time. RL has already shown success in other domains with similar or faster real-time constraints, such as robotics^36^ and energy systems^37^. To enable the transfer from simulated training environment to real-world deployment, adequate sensing of the environment is required. The RL wake steering requires anticipatory control, which depends on upstream wind field information, as shown in the closed-loop controller test, which may be realized through turbine-mounted nacelle LiDAR systems, providing upstream velocity measurements at the required frequency. This requirement could be reduced through the application of state space estimation methods^38,39^.

To approach zero-shot deployment, where the policy trained in simulation is applied directly to the physical wind farm, robust domain generalization strategies are essential. These include training across a wide ensemble of inflow conditions (e.g., wind speed, turbulence intensity, thermal stratification). Transfer to real wind farms may also benefit from online adaptation mechanisms, such as policy fine-tuning. These would require safe exploration mechanisms to ensure operational stability during learning.

Our analysis suggests further lines of inquiry for future research.

1. Constrained controller design: In this study, an emphasis was placed on not constraining the possible behavior of the controller too much, in order to allow flexibility in potential control strategies. This comes at the cost of convergence speed and computational burden. The results of our analysis suggest that a more effective way to design dynamic wind farm controllers could be to first obtain optimal static angles using Bayesian optimization, and then constrain the controller to only switch between the (finitely many) static optima obtained either directly from optimization or symmetry considerations.
2. Sensor placement: The hyperparameter sweeps conducted in this study suggest that sensor location and the number of sensors are crucial for obtaining good performance. This is important as the RL method relies on the increased level of information provided to it. A dedicated study analyzing and optimizing sensor placement and the measured fields would be beneficial. Additionally, including both biased and noisy measurements in the training, that replicate real sensor constraints, may be beneficial in transferring simulation trained controllers to real wind farm application.
3. Generalization to layout and conditions: In this study a single wind farm layout was studied under a single wind condition. It is expected that different wind farm configurations, with larger numbers of wind turbines, and different wind conditions would result in different optimal control strategies. Further work would be needed to design and train controllers that are robust to these different conditions.
4. Mechanical stresses: In the study, the reward was chosen as the instantaneous power output of the wind farm, in order to simplify convergence. In reality, moving the turbines will incur a cost in terms of both energy and mechanical stresses, which should be included in the reward design.
5. Reference power signal tracking: Although this study focused on maximizing wind farm power output, it may be beneficial for grid balancing to train the RL controller to track a reference power signal. This would be particularly valuable for understanding controller behavior when the wind farm needs to reduce its power outcome.
6. Combined induction-yaw control: Adding additional control parameters to wind turbines may allow greater flexibility for the trained controller. This may yield greater performance gains over a wider set of conditions as has been studied in static control^15,40^. Control of the induction could be included as an additional output of the controller or as blade pitch angle or the generator torque of the turbine.

## Methods

We couple high-fidelity simulations of a wind farm with an *active closed-loop controller*, which periodically updates the yaw angles of the wind turbines, in order to maximize the combined power output. LES is used to model the three-dimensional flow through a series of wind turbines operating in an atmospheric boundary layer. Periodically, we allow a closed-loop active controller to interact with the LES solver. The controller is represented as a multi-layer perceptron (MLP) and dynamically updates the yaw angles of each turbine as a function of the wind velocities in the vicinity of the turbines. The controller parameters are optimized using an RL setup, in order to maximize the total mean farm power output of the controlled system.

The wind farm configuration, inflow conditions, details of the LES solver and turbine modeling within the flow environment are described first, followed by the optimization problem and the RL framework used. The BO method used for the static and dynamic comparisons are also described. Finally we present the integration of the LES code with the RL controller and our solution that allows efficient coupling.

### Wind farm configuration

In this study a simple wind farm configuration with three turbines aligned in the *x*-direction and separated by a distance of 5*D* is considered, where the diameter of the wind turbine rotors is *D* = 126 m. The mean stream-wise velocity of the incoming flow is in the *x*-direction and has a magnitude of *U*_*∞*_ = 7.5 ms^−1^ at the wind turbine hub height, *y*_*h*_ = 90 m. In the current work, the computational domain is of size *l*_*x*_, *l*_*y*_, *l*_*z*_ = 19*D*, 4*D*, 7*D* and is discretized with a spacing *D*/10 into *n*_*x*_, *n*_*y*_, *n*_*z*_ = 193, 41, 72 grid points. This grid spacing was chosen to capture the main turbulent structures in the flow while also providing accurate results of the turbines far wake flow and power output. It is consistent with the grid spacing used in previous studies^41–44^.

### Large-Eddy simulation (LES)

Three-dimensional LES are performed using Winc3D^45^, the wind farm simulator of the finite-difference framework XCompact3D^46^. Winc3D is used to predict the aerodynamics of the flow around the turbines and to determine the resulting power output of each turbine in the wind farm. The simulations are based on the incompressible, explicitly filtered Navier-Stokes equations:

2 $$\frac{\partial \widehat{{u}_{i}}}{\partial t}+\frac{1}{2}\left(\widehat{{u}_{j}}\frac{\partial {u}_{i}}{\partial {x}_{i}}+\frac{\partial \widehat{{u}_{i}}\widehat{{u}_{j}}}{\partial {x}_{j}}\right)= -\frac{1}{\rho }\frac{\partial \widehat{p}}{\partial {x}_{i}}-\frac{\partial {\tau }_{ij}}{\partial {x}_{j}} \\ +\nu \frac{{\partial }^{2}\widehat{{u}_{i}}}{\partial {x}_{j}\partial {x}_{j}}+\frac{{F}_{i}}{\rho },\,(i=1,2,3)$$

3 $$\frac{\partial \widehat{{u}_{j}}}{\partial {x}_{j}}=0.$$

Here, spatial filtering is denoted by $\widehat{\cdot }$ and is applied to the pressure *p* and the velocity components *u*_*i*_, with the summation implied over the *j* index. Fluid density and kinematic viscosity are denoted by *ρ* and *ν*, respectively. External forces applied to the fluid (including the turbine model) are represented by *F*_*i*_, and *τ*_*ij*_ are the sub-filter scale stresses, defined as ${\tau }_{ij}=\widehat{{u}_{i}{u}_{j}}-\widehat{{u}_{i}}\widehat{{u}_{j}}$.

These stresses are modeled using the standard Smagorinsky model^47^:

4 $${\tau }_{ij}=-2{({C}_{s}\Delta )}^{2}\widehat{{S}_{ij}}| \widehat{S}| ,$$

where $\widehat{{S}_{ij}}$ is the filtered strain-rate tensor and $| \widehat{S}|$ its magnitude. The filter width *Δ* and the Smagorinsky coefficient *C*_*s*_ define the sub-filter scale viscosity. Near-wall damping is applied to *C*_*s*_ using the formulation of Mason and Thomson^48^, where:

5 $${C}_{s}={\left({C}_{0}^{n}+{\left[\kappa \frac{y+{y}_{0}}{\Delta }\right]}^{-n}\right)}^{-1/n},$$

where *C*_0_ = 1.4 is the Smagorinsky constant far from the wall, *κ* = 0.4 is the von Kármán constant, *n* = 3 the growth parameter, and *y*_0_ the surface roughness height.

Winc3D operates on a Cartesian mesh and employs high-order compact finite-difference schemes for spatial differentiation, filtering, and interpolation. Spatial derivatives are computed using sixth-order implicit schemes, while temporal integration is performed using a third-order Adams-Bashforth method. These high-order compact schemes offer quasi-spectral accuracy while allowing for non-periodic boundary conditions, enabling efficient resolution of small-scale turbulence with reduced computational cost^49^ that enables the long simulations needed for training the RL. The incompressibility of the velocity field is ensured by solving the pressure Poisson equation in spectral space using three-dimensional fast Fourier transforms (FFTs) and the concept of the modified wave number. The pressure grid is half-staggered from the velocity grid to avoid spurious pressure perturbations.

The simplicity of the Cartesian mesh is exploited with a 2D domain decomposition strategy implemented using standardized MPI that gives the code excellent strong and weak scaling properties^46,50^. The computational domain is divided into pencils, each of which is handled by an MPI process. The derivatives, interpolations, and one-dimensional FFTs in the x, y and z directions are performed within the X, Y, and Z pencils, allowing an efficient computation. Each operation is performed sequentially in one direction, with global transpositions enabling to switch from one pencil to another.

#### Actuator disk model

Rather than solving the full geometry of the wind turbines, their effect on the wind can be modeled using the actuator surface^51^, actuator line^52^ or actuator disc^53^ methods. This work uses the actuator disc model because of its relatively lower computational cost. The actuator disc model does not resolve the geometry of the individual turbine blades but instead uses a porous disc representation over their swept area. Although small-scale flow structures are not captured in the wake, the work of Revaz and Porté-Agel^42^ showed that the actuator disc model can provide accurate results of the far wake flow, as well as wind farm thrust and power predictions. More detailed actuator line or surface models may offer better resolution in the near wake but considerably increase the simulation cost.

The implementation of the actuator disk model used here is detailed and validated in Bempedelis et al.^54^, based on the method of Calaf et al.^9^ using the 1D momentum theory. A uniform drag force is imparted on the flow over the swept area of the wind turbine blades, *A*, defined by,

6 $${F}_{t}=-\frac{1}{2}\,\rho \,A\,{C}_{T}{\left\langle \frac{{U}_{r}}{(1-a)}\right\rangle }^{2},$$

where $\left\langle \ldots \right\rangle$ denotes an averaging over the actuator disc and *U*_*r*_ is the rotor normal velocity. The axial induction factor, *a*, is derived from the thrust coefficient, *C*_*T*_, such that $a=\frac{1}{2}\left(1-\sqrt{1-{C}_{T}}\right)$. This force is distributed over the disc area with a super-Gaussian smoothing applied, as described in King et al.^55^. The power output from the turbine can then be calculated as,

7 $$P=-{F}_{t}{U}_{r}.$$

#### Atmospheric boundary layer (ABL)

To generate the inflow conditions for the LES wind farm environments, precursor simulations are conducted to generate atmospheric boundary layers. The atmospheric boundary layer is generated using a friction velocity u^*^ = 0.442 ms^−1^, a boundary layer height *δ* = 504*m*, and a roughness length *z*_0_ = 0.05*m*. These parameters were chosen to match those used in previous studies in Bempedelis et al.^43,56^ that best match the wind speed and turbulence intensity conditions observed and reported by Barthelmie et al.^57^ that are representative of offshore wind conditions. At each simulation step, a 2D slice of the flow field, orientated normal to the stream-wise direction, is stored to be used as the inflow condition for the wind farm simulations. To minimize spurious low-frequency periodicity introduced by the recycling method in the precursor simulation, the domain was extended to a physical length of 224*D* = 28, 224 m. This helps to avoid artificial peaks in the frequency of the inflow caused by repeated large-scale structures. Additionally, at each recycling step, the inflow plane is laterally shifted by one-eighth of the domain width to de-correlate repeating features^58^. These modifications were necessary to prevent the RL controller from exhibiting phase-locked behavior in response to periodic artifacts in the inlet when using shorter precursor domains, ensuring instead that it responds to the broadband turbulent features characteristic of the atmospheric boundary layer.

### Reinforcement learning

RL is a machine learning approach that enables a controller (referred to as an agent in the RL literature) to optimize decision making by interacting with an environment, guided by a reward signal. In this section, we explain how we cast the wind farm optimization problem within the RL framework. We first outline the fundamental concepts of RL.

#### Fundamentals of RL

In RL, the environment is typically modeled as a discrete-time Markov decision process^19^, defined by the tuple $({{\mathcal{S}}},{{\mathcal{A}}},{{\mathcal{P}}},{{\mathcal{R}}})$. Here, ${{\mathcal{S}}}$ denotes the set of states representing possible configurations of the system, ${{\mathcal{A}}}$ is the set of admissible actions available to the agent, and ${{\mathcal{P}}}({s}^{{\prime} }| s,a)$ is the state transition probability function, which describes the likelihood of transitioning to state ${s}^{{\prime} }$ given that the agent takes action *a* in state *s*. The reward function, ${{\mathcal{R}}}(s,a)$, assigns an immediate reward based on the chosen action and the resulting state.

A *policy *$\pi :{{\mathcal{S}}}\to {{\mathcal{A}}}$ prescribes the agent’s decision-making strategy by mapping states to actions. More generally, a stochastic policy defines a probability distribution over actions, *π*(*a*∣*s*), from which the agent samples its next move. Starting from an initial state *s*_0_, the agent sequentially selects actions based on the policy, generating a trajectory or *rollout *${\bar{s}}_{\pi }=({s}_{0},{a}_{0},{s}_{1},{a}_{1},{r}_{1},\ldots ,{s}_{k},{a}_{k},{r}_{k})$, where:

8 $${a}_{k} \sim \pi (\cdot | {s}_{k}), \, \, {r}_{k}={{\mathcal{R}}}({s}_{k},{a}_{k}), \, \, {s}_{k+1} \sim {{\mathcal{P}}}(\cdot | {s}_{k},{a}_{k}).$$

The performance of a given policy is quantified by the *return*, defined as the discounted sum of rewards along the trajectory:

9 $$R({\bar{s}}_{\pi })={\sum }_{k=0}^{L}{\gamma }^{k}{r}_{k},$$

where 0 < *γ*≤1 is the discount factor that determines the relative importance of immediate versus future rewards and *L* > 0 is the duration of an episode. The objective of RL is to identify the optimal policy, π^*^, that maximizes the expected return:

10 $${\pi }^{* }={{{\rm{argmax}}} }_{\pi }{{\mathbb{E}}}_{{s}_{0}}[{R}_{\pi }].$$

The role of the parameter *γ* is to regularize the sum in Eq. (9). Since ${\sum }_{k=0}^{\infty }{\gamma }^{k}={(1-\gamma )}^{-1}$ whenever 0 < *γ* < 1, it follows that the implication of choosing *γ* < 1 is to effectively truncate the policy return to a finite future time horizon of (1−*γ*)^−1^ time steps. In practice, the $\arg \max$ in Eq. (10) is intractable, and instead the policy is parametrized as a deep neural network with parameters *θ*, which are learned through gradient descent.

#### Soft actor critic (SAC)

Various RL algorithms have been developed to iteratively converge to an optimal policy as defined in Eq. (10). Foundational methods include Q-Learning^59,60^, policy iteration^61^, temporal difference learning^62^, Double Q-Learning^63^, Deep Deterministic Policy Gradient^64^, Twin Delayed Deep Deterministic Policy Gradient^65^ and Proximal Policy Optimization^34^. RL algorithms are notoriously sample inefficient, often requiring on the order of 10^5^ − 10^7^ environment interactions to reach convergence^34,66^. Overestimation bias and the exploration-exploitation trade-off can lead to the discovery of suboptimal policies and lack of stability during training. In addition, catastrophic forgetting can lead to policies which suddenly ‘unlearn’ previously mastered tasks^67,68^.

**Fig. 7:**
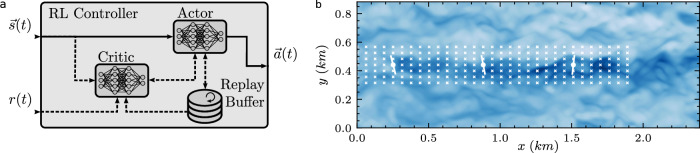
Reinforcement learning controller architecture and flow sensing configuration. **a** Diagram showing the soft actor-critic controller algorithm with the dashed arrows showing the flow of information during training only and the solid arrows during training and evaluation. **b** Location of the velocity sensors around the turbines shown in an *x* − *z* slice at the turbine hub height. The sensor locations are shown by the white crosses. The observations include the stream-wise velocity component as input to the RL policy, the instantaneous field of which is shown by the contours.

Soft Actor Critic (SAC) is a state-of-the-art RL method for continuous control problems^69^. SAC is a model-free RL algorithm without policies, which separates the policy (the actor) from the estimation of the expected return (the critic) as seen in (Fig. 7a), leading to improved stability of convergence and reducing the risk of overestimating the value of actions. SAC extends traditional actor-critic methods by incorporating an entropy regularization term that balances the trade-off between exploration and exploitation into the objective. The off-policy nature of SAC allows it to make use of an experience replay buffer, leading to higher sample efficiency compared to on-policy RL methods. The off-policy nature of SAC in addition permits the use of parallel experience collection. We simulate *M* = 32 environments in parallel during training and *M* = 16 environments during evaluation, decreasing the wall-clock time of the optimization loop.

Our implementation of SAC is based on the PyTorch machine learning library^70^ and makes use of the TorchRL library for RL^71^.

#### States, actions and rewards for wind farm optimization

In order to cast our wind farm optimization problem in the RL framework, we define a discrete time step of *dt*^RL^ = 10 s. The LES solver interacts with the RL controller every *dt*^RL^ time units, so that one discrete step of the RL rollout corresponds to 50 discrete steps of the LES solver. At each discretized time step, we update the yaw angles $\vec{\alpha }=({\alpha }_{1}(t),{\alpha }_{2}(t),{\alpha }_{3}(t))$ of the individual turbines. We employ a differential policy, which outputs the angular velocity with which each turbine moves. This is done to ensure that the simulation remains numerically stable, as rapid changes in the yaw angle of the turbines are likely to lead to numerical issues and be impractical to perform with a real wind turbine. The angles at time step *t* are therefore updated as,

11 $$\begin{array}{c}{\alpha }_{n}(t)={\alpha }_{n}(t-d{t}^{{{\rm{RL}}}})+{v}_{n}(t)\,d{t}^{{{\rm{RL}}}},\,n=1,\ldots ,3,\\ \vec{a}(t)=\vec{\alpha }(t)=\vec{\alpha }(t-d{t}^{{{\rm{RL}}}})+\vec{v}(t)\,d{t}^{{{\rm{RL}}}},\end{array}$$

where $\vec{v}(t)=({v}_{1}(t),{v}_{2}(t),{v}_{3}(t))$ is the vector of angular velocities of the turbines, which is the output of the policy *π*. We limit the angular velocity to a maximum of $\left|{v}_{n}\right|\le 1.0$ deg *s*^−1^. In addition, we limit the maximum yaw angle to $\left|{\alpha }_{n}\right|\le 4{0}^{\circ }$ symmetrically.

The flow field is sensed through a total of 231 velocity sensors arranged in a 2*D* plane at hub height surrounding the turbines, see Fig. 7b. The number of sensors was chosen as a result of a parameter sweep (see more details in the Supplementary Table 1 and Fig. 4). We only use the stream-wise velocity component *u*_*x*_, which is the most informative (least noisy) of the three velocity components (*u*_*x*_, *u*_*y*_, *u*_*z*_). The velocities of each individual sensor are averaged over the course of the discrete time step *dt*^RL^ to smooth the signal. The averaged sensor observations form the observation vector $\vec{o}(t)$. We then define the state vector $\vec{s}(t)$ as,

12 $$\vec{s}(t)=\left(\vec{o}(t),\vec{\alpha }\left(t-d{t}^{{{\rm{RL}}}}\right)\right),$$

where $\vec{\alpha }(t-d{t}^{{{\rm{RL}}}})$ is the vector of turbine yaw angles from the previous time step. The state vector $\vec{s}(t)$ forms the input of the RL policy *π*.

The reward is mainly composed of the instantaneous total wind farm power output. The LES solver provides the instantaneous power output of each individual turbine, denoted as *P*_*n*_(*t*) for the *n*-th turbine for *n* = 1, …, 3. The instantaneous total power output of the wind farm can then be calculated as,

13 $$P(t)={\sum }_{n=1}^{3}{P}_{n}(t).$$

In addition, we add a large angle penalty term, which prevents the controller from converging to very large yaw angles:

14 $$r(t)=P(t)-\frac{\lambda }{3}{\sum }_{n=1}^{3}{\left(\frac{{\alpha }_{n}(t)}{{\alpha }_{\max }}\right)}^{\kappa },$$

where *P* is defined in Eq. (13) and *λ* and *κ* are hyperparameters; see Supplementary Table 1. By integrating (13) in time, we can obtain the mean wind farm power output over time,

15 $$\bar{P}={lim}_{T\to \infty }\frac{1}{T} \int _{0}^{T}P(t)\,dt.$$

We note that, if *γ* = 1 and *λ* = 0, then ${lim}_{L\to \infty }\frac{1}{L}{\sum }_{k=0}^{L}{\gamma }^{k}{r}_{k}=\bar{P}$. Therefore, for this choice of hyperparameters, maximizing the return *R*_*π*_ for a sufficiently long rollout is equivalent to maximizing the mean farm power $\bar{P}$. In practice, we use *γ* < 1, making the return a regularized (or truncated) version of the mean farm power.

### Bayesian optimization

To establish a static baseline for comparison with the RL controller, we also perform BO. In contrast to RL, which frames the control as a Markov decision process and learns a dynamic policy based on the observed system states over a series of time steps, BO identifies fixed yaw angles that maximize the mean wind farm power output:

16 $${\vec{\alpha }}_{BO}={{{\rm{argmax}}} }_{{\vec{\alpha }}}{\sum }_{n=1}^{N}\overline{P}(\vec{\alpha })\,\,\,\,\,{{\rm{subject}}}\; {{\rm{to}}}\,\,\,\vec{\alpha }\in [-{\alpha }_{b},\,{\alpha }_{b}].$$

Bayesian optimization is an efficient sequential design strategy for global optimization of expensive to evaluate functions that uses a surrogate model to guide the search^72–74^. The model is iteratively updated by adding new data at locations defined by an acquisition function.

In this work, the optimization is carried out using a Gaussian process surrogate model^75^ with a Matérn 5/2 kernel and automatic relevance determination and an upper confidence bound (UCB)^76^ acquisition function implemented with BoTorch^77^. The UCB exploration parameter is initialized at *β* = 4 and annealed linearly throughout the optimization. The search space covers the yaw angles of all three turbines, with bounds defined by *α*_*b*_ = 40 for each. The optimization is initialized with 6 Latin hypercube samples and proceeds for 50 BO iterations. Each optimization iteration consists of an ensemble of 32 LES, each averaged over 5000 s of simulated time to produce $\overline{P}$. This configuration substantially increases computational cost but reduces the likelihood of premature convergence or noise from time averaging errors in the estimated optimum.

### Dynamic Bayesian optimization

To establish a baseline for dynamic control, we also implement a dynamic BO controller based on the incoming wind angle. Instead of identifying a single set of fixed yaw angles that maximize the mean farm power under a prescribed inflow direction as in the static BO approach, the dynamic BO method seeks to approximate the optimal yaw settings as a function of the instantaneous mean wind direction. This mapping is then used online within the LES environment to provide a controller that changes based on the incoming instantaneous wind direction.

For the inflow wind directions directions, *θ* ∈ [ − 10^∘^,  10^∘^] at increments of 0. 5^∘^, we independently solve the BO problem describe din Eq. (16). As in the static case, the optimization employs a Gaussian-process surrogate model with a Matérn 5/2 kernel and a UCB acquisition function.

Because running LES for all wind directions and optimization iterations would be computationally prohibitive, the BO for the dynamic controller is performed using the Gauss Curl Hybrid (GCH) wake model. This optimization results in a lookup table:

17 $$\theta \,\mapsto \,{\vec{\alpha }}_{BO}(\theta ),$$

that specifies the optimized turbine yaw settings as a function of the mean inflow direction. During an LES evaluation, this lookup table defines the dynamic BO policy.

To apply this policy to the LES environment, we place a wind direction sensor 2*D* upstream of the first turbine. At update intervals matching the RL control frequency, we compute the mean wind direction at the sensor location since the previous update and use it to query the BO lookup table for the corresponding optimal yaw angles. Finally the resulting angular velocity of the turbine yaws between updates are limited to a maximum of $\left|{v}_{n}\right|\le 1.0$ deg *s*^−1^, as is done in the RL case.

### Coupling the RL controller with LES solver

Coupling the high-fidelity LES solver with the RL controller is challenging due to the different programming languages used. While the LES solver is based on XCompact3D and is implemented in modern Fortran, the RL controller is implemented in Python using the PyTorch machine learning library. At every RL time step (corresponding to 50 LES solver steps), the controller processes the sensor observations computed by the LES solver, outputs updated yaw angles and communicates these updated angles to the LES solver. This necessitates both the solver and the RL controller to frequently ‘wait’ for the corresponding other code to compute a result before continuing.

**Fig. 8:**
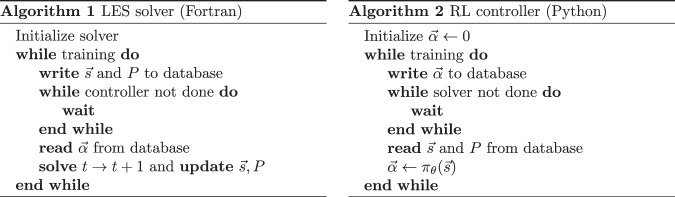
Communication loop for the large eddy simulation (LES) solver (l*eft) and RL controller (right*). Here $\vec{s}$ denotes the state (velocity sensors and previous yaw angles) and *P* denotes the wind farm power. Both codes are executed asynchronously on separate nodes and communicate with a separate database node. Synchronization is achieved by waiting for the respective counterpart to update a certain entry in the database. We have omitted the training part of the RL code to highlight the communication loop. During training, the entire loop is paused, and the parameters of the policy *π*_*θ*_ are updated. In practice, we use *M* = 32 parallel instances of the LES environment during training and *M* = 16 parallel instances during evaluation. Each instance is run on a single node of ARCHER2 (128 CPU cores).

We integrate the LES solver and RL controller components through the SmartSim library^78^, an infrastructure framework that enables the integration of machine learning workflows into traditional HPC workloads. In the SmartSim setup, the solver and controller are executed asynchronously on separate nodes and communicate with each other using SmartRedis through an additional orchestration and database node. This approach is similar to previous work combing turbulent flow control with RL on HPC^24^. We give a pseudocode outline of the system in Fig. 8.

## Supplementary information

Supplementary Information

## Data Availability

The datasets generated during the study are available on Zenodo at 10.5281/zenodo.15705117^79^.

## Code availability

The LES code XCompact3d used for the simulations is available at https://github.com/xcompact3d/Incompact3d. The code responsible for the RL and coupling with the LES is available at https://github.com/admole/Wind-RL.

## References

[1] International Energy Agency. Net zero by 2050: A roadmap for the global energy sector https://www.iea.org/reports/net-zero-by-2050 (2021).

[2] Barthelmie, R. J. & Jensen, L. E. Evaluation of wind farm efficiency and wind turbine wakes at the Nysted offshore wind farm. *Wind Energy* **13**, 573–586 (2010).

[3] Howland, M. F. et al. Collective wind farm operation based on a predictive model increases utility-scale energy production. *Nat. Energy* **7**, 818–827 (2022).

[4] Harrison-Atlas, D., Glaws, A., King, R. N. & Lantz, E. Artificial intelligence-aided wind plant optimization for nationwide evaluation of land use and economic benefits of wake steering. *Nat. Energy* **9**, 735–749 (2024).

[5] Veers, P. et al. Grand challenges in the design, manufacture, and operation of future wind turbine systems. *Wind Energy Sci.* **8**, 1071–1131 (2023).

[6] Jensen, N.*A note on wind generator interaction*. No. 2411 in Risø-M (Risø National Laboratory, 1983).

[7] Jiménez, Á, Crespo, A. & Migoya, E. Application of a LES technique to characterize the wake deflection of a wind turbine in yaw. *Wind Energy* **13**, 559–572 (2010).

[8] Bastankhah, M. & Porté-Agel, F. A new analytical model for wind-turbine wakes. *Renew. Energy* **70**, 116–123 (2014).

[9] Calaf, M., Meneveau, C. & Meyers, J. Large eddy simulation study of fully developed wind-turbine array boundary layers. *Phys. Fluids* **22**, 015110 (2010).

[10] Meyers, J. & Meneveau, C. Optimal turbine spacing in fully developed wind farm boundary layers. *Wind Energy* **15**, 305–317 (2012).

[11] Meyers, J. et al. Wind farm flow control: Prospects and challenges. *Wind Energy Sci.* **7**, 2271–2306 (2022).

[12] Mole, A. & Laizet, S. Multi-fidelity Bayesian optimisation of wind farm wake steering using wake models and large Eddy simulations. *Flow Turbulence Combust.* **115**, 1209–1234 (2025). 10.1007/s10494-024-00629-0 PMC12508008 41079150

[13] Houck, D. R. Review of wake management techniques for wind turbines. *Wind Energy* **25**, 195–220 (2022).

[14] Goit, J. P. & Meyers, J. Optimal control of energy extraction in wind-farm boundary layers. *J. Fluid Mech.* **768**, 5–50 (2015).

[15] Munters, W. & Meyers, J. Dynamic strategies for yaw and induction control of wind farms based on large-Eddy simulation and optimization. *Energies* **11**, 177 (2018).

[16] Frederik, J. A., Doekemeijer, B. M., Mulders, S. P. & Van Wingerden, J.-W. The helix approach: Using dynamic individual pitch control to enhance wake mixing in wind farms. *Wind Energy* **23**, 1739–1751 (2020).

[17] Howland, M. F., Ghate, A. S., Lele, S. K. & Dabiri, J. O. Optimal closed-loop wake steering – Part 1: Conventionally neutral atmospheric boundary layer conditions. *Wind Energy Sci.* **5**, 1315–1338 (2020).

[18] Boersma, S., Doekemeijer, B., Vali, M., Meyers, J. & Van Wingerden, J.-W. A control-oriented dynamic wind farm model: WFSim. *Wind Energy Sci.* **3**, 75–95 (2018).

[19] Sutton, R. S. & Barto, A. G. Reinforcement learning: An introduction. *MIT Press* (2018).

[20] Mnih, V. et al. Human-level control through deep reinforcement learning. *nature* **518**, 529–533 (2015). 25719670 10.1038/nature14236

[21] Mnih, V. et al. Asynchronous methods for deep reinforcement learning. In *International conference on machine learning*, 1928–1937 (PmLR, 2016).

[22] Chatzimanolakis, M., Weber, P. & Koumoutsakos, P. Learning in two dimensions and controlling in three: Generalizable drag reduction strategies for flows past circular cylinders through deep reinforcement learning. *Phys. Rev. Fluids* **9**, 043902 (2024).

[23] Xia, C., Zhang, J., Kerrigan, E. C. & Rigas, G. Active flow control for bluff body drag reduction using reinforcement learning with partial measurements. *J. Fluid Mech.* **981**, A17 (2024).

[24] Font, B., Alcántara-Ávila, F., Rabault, J., Vinuesa, R. & Lehmkuhl, O. Deep reinforcement learning for active flow control in a turbulent separation bubble. *Nat. Commun.* **16**, 1422 (2025). 39915442 10.1038/s41467-025-56408-6 PMC11802851

[25] Vignon, C., Rabault, J. & Vinuesa, R. Recent advances in applying deep reinforcement learning for flow control: Perspectives and future directions. *Phys. Fluids* **35**, 031301 (2023).

[26] Bui, V.-H., Nguyen, T.-T. & Kim, H.-M. Distributed operation of wind farm for maximizing output power: A multi-agent deep reinforcement learning approach. *IEEE Access* **8**, 173136–173146 (2020).

[27] He, B. et al. Ensemble-based Deep Reinforcement Learning for robust cooperative wind farm control. *Int. J. Electr. Power Energy Syst.* **143**, 108406 (2022).

[28] Padullaparthi, V. R., Nagarathinam, S., Vasan, A., Menon, V. & Sudarsanam, D. FALCON- FArm Level CONtrol for wind turbines using multi-agent deep reinforcement learning. *Renew. Energy* **181**, 445–456 (2022).

[29] Dong, H., Zhang, J. & Zhao, X. Intelligent wind farm control via deep reinforcement learning and high-fidelity simulations. *Appl. Energy* **292**, 116928 (2021).

[30] Wang, H., He, S., Yan, J., Han, S. & Liu, Y. Deep reinforcement learning-driven wind farm flow control considering dynamic wind. *Energy Convers. Manag.* **337**, 119888 (2025).

[31] Liew, J., Göçmen, T., Lio, W. H. & Larsen, G. C. Model-free closed-loop wind farm control using reinforcement learning with recursive least squares. *Wind Energy* **27**, 1173–1187 (2024).

[32] Monroc, C. B., Bušić, A., Dubuc, D. & Zhu, J. Wfcrl: A multi-agent reinforcement learning benchmark for wind farm control. *Adv. Neural Inf. Process. Syst.* **37**, 133254–133281 (2024).

[33] Korb, H., Asmuth, H., Stender, M. & Ivanell, S. Exploring the application of reinforcement learning to wind farm control. *J. Phys. Conf. Ser.* **1934**, 012022 (2021).

[34] Schulman, J., Wolski, F., Dhariwal, P., Radford, A. & Klimov, O. Proximal policy optimization algorithms. *arXiv preprint arXiv:1707.06347* (2017).

[35] Beckett, G. et al. Archer2 service description 10.5281/zenodo.14507040 (2024).

[36] Kober, J., Bagnell, J. A. & Peters, J. Reinforcement learning in robotics: A survey. * Int. J. Robot. Res.* **32**, 1238–1274 (2013).

[37] Degrave, J. et al. Magnetic control of tokamak plasmas through deep reinforcement learning. *Nature* **602**, 414–419 (2022). 35173339 10.1038/s41586-021-04301-9 PMC8850200

[38] Erichson, N. B. et al. Shallow neural networks for fluid flow reconstruction with limited sensors. *Proc. R. Soc. A: Math., Phys. Eng. Sci.* **476**, 20200097 (2020). 10.1098/rspa.2020.0097 PMC7428025 32831593

[39] Fukami, K. & Taira, K. Grasping extreme aerodynamics on a low-dimensional manifold. *Nat. Commun.* **14**, 6480 (2023). 37838743 10.1038/s41467-023-42213-6 PMC10576750

[40] Heck, K. S., Liew, J., Upfal, I. M. L. & Howland, M. F. Joint Yaw-Induction Control Optimization for Wind Farms (2025).

[41] Calaf, M., Meneveau, C. & Parlange, M. Large eddy simulation study of a fully developed thermal wind-turbine array boundary layer. In Kuerten, H., Geurts, B., Armenio, V. & Fröhlich, J. (eds.) *Direct and Large-Eddy Simulation VIII*, 239–244 (Springer Netherlands, Dordrecht, 2011).

[42] Revaz, T. & Porté-Agel, F. Large-Eddy simulation of wind turbine flows: A new evaluation of actuator disk models. *Energies* **14**, 3745 (2021).

[43] Bempedelis, N., Gori, F., Wynn, A., Laizet, S. & Magri, L. Data-driven optimisation of wind farm layout and wake steering with large-eddy simulations. *Wind Energy Sci.* **9**, 869–882 (2024).

[44] Jané-Ippel, C., Bempedelis, N., Palacios, R. & Laizet, S. Bayesian optimisation of a two-turbine configuration around a 2D hill using large Eddy simulations. *Wind Energy* **27**, 1412–1426 (2024).

[45] Deskos, G., Laizet, S. & Palacios, R. WInc3D: A novel framework for turbulence-resolving simulations of wind farm wake interactions. *Wind Energy* **23**, 779–794 (2020).

[46] Bartholomew, P. et al. Xcompact3d: An open-source framework for solving turbulence problems on a cartesian mesh. *SoftwareX* **12**, 100550 (2020).

[47] Smagorinsky, J. General ciculation experiments with the primitive equa- tions. *Mon. Weather Rev.* **91**, 99–164 (1963).

[48] Mason, P. J. & Thomson, D. J. Stochastic backscatter in large-eddy simulations of boundary layers. *J. Fluid Mech.* **242**, 51–78 (1992).

[49] Laizet, S. & Lamballais, E. High-order compact schemes for incompressible flows: A simple and efficient method with quasi-spectral accuracy. *J. Comput. Phys.* **228**, 5989–6015 (2009).

[50] Laizet, S. & Li, N. Incompact3d: A powerful tool to tackle turbulence problems with up to computational cores. *Int. J. Numer. Methods Fluids* **67**, 1735–57 (2011).

[51] Shen, W. Z. & Nørk, J. Actuator Surface Model for Wind Turbine Flow Computations. In *Proceedings of European Wind Energy Conference 2007* (2007).

[52] Sørensen, J. N. & Shen, W. Z. Numerical modeling of wind turbine wakes. *J. Fluids Eng.* **124**, 393–399 (2002).

[53] Mikkelsen, R.* Actuator Disc Methods Applied to Wind Turbines*. Ph.D. thesis, Technical University of Denmark (2004).

[54] Bempedelis, N., Laizet, S. & Deskos, G. Turbulent entrainment in finite-length wind farms. *J. Fluid Mech.* **955**, A12 (2023).

[55] King, R. N., Dykes, K., Graf, P. & Hamlington, P. E. Optimization of wind plant layouts using an adjoint approach. *Wind Energy Sci.* **2**, 115–131 (2017).

[56] Wu, Y.-T. & Porté-Agel, F. Modeling turbine wakes and power losses within a wind farm using LES: An application to the Horns Rev offshore wind farm. *Renew. Energy* **75**, 945–955 (2015).

[57] Barthelmie, R. J. et al. Modelling and measuring flow and wind turbine wakes in large wind farms offshore. *Wind Energy* **12**, 431–444 (2009).

[58] Munters, W., Meneveau, C. & Meyers, J. Shifted periodic boundary conditions for simulations of wall-bounded turbulent flows. *Phys. Fluids* **28**, 025112 (2016).

[59] Watkins, C. J. C. H. Learning from delayed rewards. *Cambridge: Cambridge University* (1989).

[60] Watkins, C. J. & Dayan, P. Q-learning. *Mach. Learn.* **8**, 279–292 (1992).

[61] Howard, R. A. Dynamic programming and markov processes. *MIT Press* **2**, 39–47 (1960).

[62] Sutton, R. S. Learning to predict by the methods of temporal differences. *Mach. Learn.* **3**, 9–44 (1988).

[63] Van Hasselt, H., Guez, A. & Silver, D. Deep reinforcement learning with double q-learning. In *Proceedings of the AAAI conference on artificial intelligence*, **30** (2016).

[64] Lillicrap, T. Continuous control with deep reinforcement learning. *arXiv preprint arXiv:1509.02971* (2015).

[65] Fujimoto, S., Hoof, H. & Meger, D. Addressing function approximation error in actor-critic methods. In *International conference on machine learning*, 1587–1596 (PMLR, 2018).

[66] Mnih, V. Playing atari with deep reinforcement learning. *arXiv preprint arXiv:1312.5602* (2013).

[67] Zhang, T., Wang, X., Liang, B. & Yuan, B. Catastrophic interference in reinforcement learning: A solution based on context division and knowledge distillation. *IEEE Trans. Neural Netw. Learn. Syst.* **34**, 9925–9939 (2022). 10.1109/TNNLS.2022.3162241 35439142

[68] Hafez, M. B., Immisch, T., Weber, T. & Wermter, S. Map-based experience replay: a memory-efficient solution to catastrophic forgetting in reinforcement learning. *Front. Neurorobotics* **17**, 1127642 (2023). 10.3389/fnbot.2023.1127642 PMC10333526 37440981

[69] Haarnoja, T., Zhou, A., Abbeel, P. & Levine, S. Soft actor-critic: Off-policy maximum entropy deep reinforcement learning with a stochastic actor. In *International conference on machine learning*, 1861–1870 (PMLR, 2018).

[70] Paszke, A. et al. Pytorch: An imperative style, high-performance deep learning library. *Advances in neural information processing systems* **32** (2019).

[71] Bou, A. et al. Torchrl: A data-driven decision-making library for pytorch (2023). 2306.00577.

[72] Jones, D. R., Schonlau, M. & Welch, W. J. Efficient global optimization of expensive black-box functions. *J. Glob. Optim.* **13**, 455–492 (1998).

[73] Snoek, J., Larochelle, H. & Adams, R. P. Practical bayesian optimization of machine learning algorithms. In Pereira, F., Burges, C., Bottou, L. & Weinberger, K. (eds.) *Advances in Neural Information Processing Systems*, **25** (Curran Associates, Inc., 2012).

[74] Shahriari, B., Swersky, K., Wang, Z., Adams, R. P. & de Freitas, N. Taking the Human Out of the Loop: A Review of Bayesian Optimization. *Proc. IEEE* **104**, 148–175 (2016).

[75] Rasmussen, C. E. & Williams, C. K. I.*Gaussian Processes for Machine Learning*. Adaptive Computation and Machine Learning (MIT Press, Cambridge, Mass., 2008), 3. print edn.

[76] Srinivas, N., Krause, A., Kakade, S. & Seeger, M. Gaussian Process Optimization in the Bandit Setting: No Regret and Experimental Design. *Proceedings of the 27th International Conference on International Conference on Machine Learning*, 1015–1022 (2009).

[77] Balandat, M. et al. Botorch: A framework for efficient Monte-Carlo Bayesian optimization. Advances in neural information processing systems **33**:21524–21538 (2020).

[78] Partee, S. et al. Using machine learning at scale in numerical simulations with smartsim: An application to ocean climate modeling. *J. Comput. Sci.* **62**, 101707 (2022).

[79] Mole, A., Weissenbacher, M., Rigas, G. & Laizet, S. Dataset for paper: Reinforcement learning increases wind farm power production by enabling closed-loop collaborative control 10.5281/zenodo.15705117 (2025). 10.1038/s44172-026-00667-8 PMC13350969 42086930

