# Supplementary material for: Reinforcement learning increases wind farm power production by enabling closed-loop collaborative control
Source: Commun Eng. 2026 May 5;5:129. doi: 10.1038/s44172-026-00667-8 (PMC13350969; doi:10.1038/s44172-026-00667-8)
Supplement: Supplementary file 1 — Supplementary Information [file 44172_2026_667_MOESM1_ESM.pdf]

Supplementary Information for

Reinforcement Learning Increases Wind Farm Power Production by Enabling Closed-Loop Collaborative Control

Andrew Mole[1], Max Weissenbacher[1], Georgios Rigas[1], Sylvain Laizet[1]
[1]Department of Aeronautics, Imperial College London, London, UK

Supplementary Figure: Dynamic BO Lookup Table

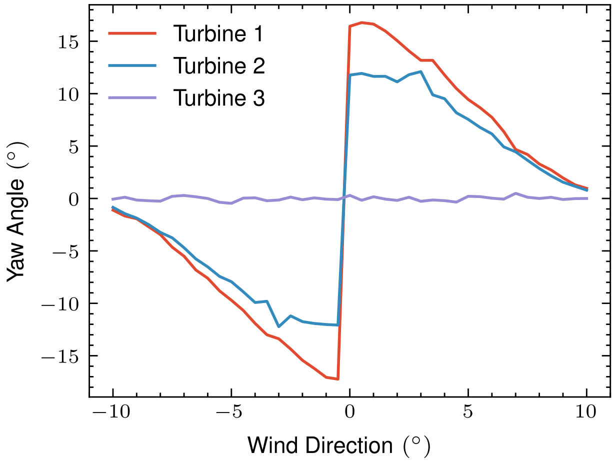

Figure S1: Optimal yaw angles for the three wind turbines calculated across a range of mean inflow directions calculated with independent Bayesian optimization (BO) runs using an analytical wake model. These results are used in the dynamic BO controllers lookup table to provide desired yaw angle updates for instantaneous wind directions.

Supplementary Figure: Time Lag Correction

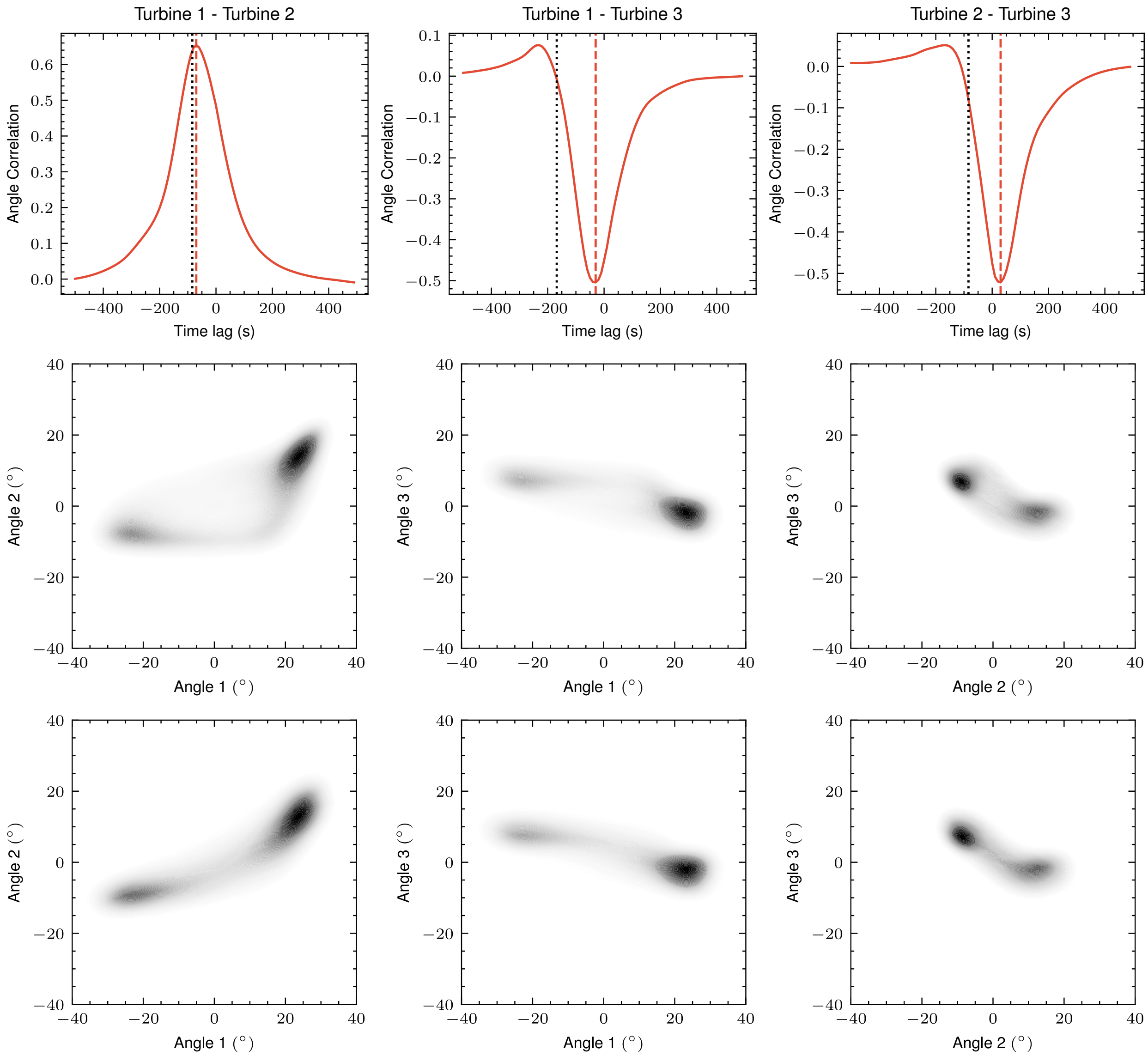

Figure S2: Cross-correlation analysis of yaw angle pairs. Kendall's tau cross-correlation functions are shown for *(a)* Angle 1 – Angle 2, *(b)* Angle 1 – Angle 3, and *(c)* Angle 2 – Angle 3 pairs across a range of time lags. The red dashed vertical line indicates the lag corresponding to the peak cross-correlation, highlighting the characteristic response time between variables. The black dotted vertical line marks the expected advection time for each pair, providing a reference for physical transport processes. *(d-f)* Joint probability density plots of angle pairs before correcting for time shifts and *(g-i)* after correcting for time-shifts determined from the cross-correlation analysis. Each plot shows the distribution of observed angle combinations for the respective variable pairs. Time shift correction results in increased density along the diagonal, reflecting better synchronization between yaw angles.

Supplementary Information: Yaw Angle Correlation Linear Models

The fitted models, shown in Fig. 3(c)(d)(e) are given by:

$$\alpha_2 = (0.2747 \pm 0.0083) + (0.4492 \pm 0.0004)\,\alpha_1\,, \tag{1}$$
$$\alpha_3 = (3.2572 \pm 0.0061) + (-0.2269 \pm 0.0003)\,\alpha_1\,, \tag{2}$$
$$\alpha_3 = (3.1932 \pm 0.0061) + (-0.4256 \pm 0.0006)\,\alpha_2\,, \tag{3}$$

where we indicate the fitted parameters as $m{\pm}s$ with m the estimated parameter and s its estimated standard error.

Supplementary Figure: Barycenter Distribution

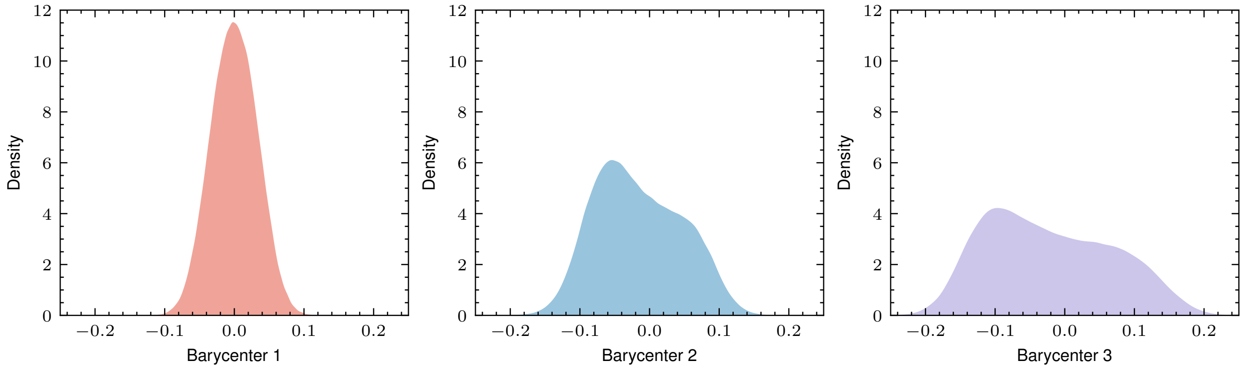

Figure S3: Probability density functions (PDFs) of the stream-wise velocity barycenter positions for three locations upstream of *(a)* Turbine 1, *(b)* Turbine 2 and *(c)* Turbine 3. PDFs are calculated using kernel density estimation with a Gaussian kernel and bandwidth selected according to Scott's rule. The barycenter position represents the spatial centroid of the velocity deficit region, providing a statistical description of upstream flow structures influencing turbine inflow. In front of Turbine 1 the flow is statistically symmetrical with asymmetry introduced downstream by the reinforcement learning yaw control.

Supplementary Table: Reinforcement Learning Hyperparameters

Table S1: Values of the relevant SAC hyperparameters and wind farm environment parameters. The column 'Training' contains the values used for the final training runs. The column 'Sweeps' contains either the standard value used, or the range of values considered in hyperparameter sweeps.

Parameter	Training	Sweeps
Discount factor γ	0.99	$[0.8, 0.99]$
Learning rate (actor and critic)	3×10^{-6}	$[3 \times 10^{-6}, 3 \times 10^{-4}]$
Learning rate (entropy)	1×10^{-5}	$[1 \times 10^{-6}, 3 \times 10^{-4}]$
Initial entropy loss multiplier	10	$[1, 100]$
Optimizer batch size	256	256
Frames per batch	512	512
Number of probes per turbine	77	$[10, 77]$
Initial random steps	$3.2 \times 10^5 s$	$3.2 \times 10^5 s$
Episode length	$5 \times 10^3 s$	$5 \times 10^3 s$
Number of episodes	2000	600
Number of parallel environments	32	32
Maximum yaw angle $\alpha_{\max}$	40 deg	40 deg
Maximum yaw velocity $v_{\max}$	$1.0\,\mathrm{deg}\,s^{-1}$	$1.0\,\mathrm{deg}\,s^{-1}$
λ (reward parameter)	0.5	0.5
κ (reward parameter)	26	26
dt_{RL}	$10s$	$10s$
dt_{LES}	$0.2s$	$0.2s$

Supplementary Figure: Reinforcement Learning Hyperparameter Sweeps

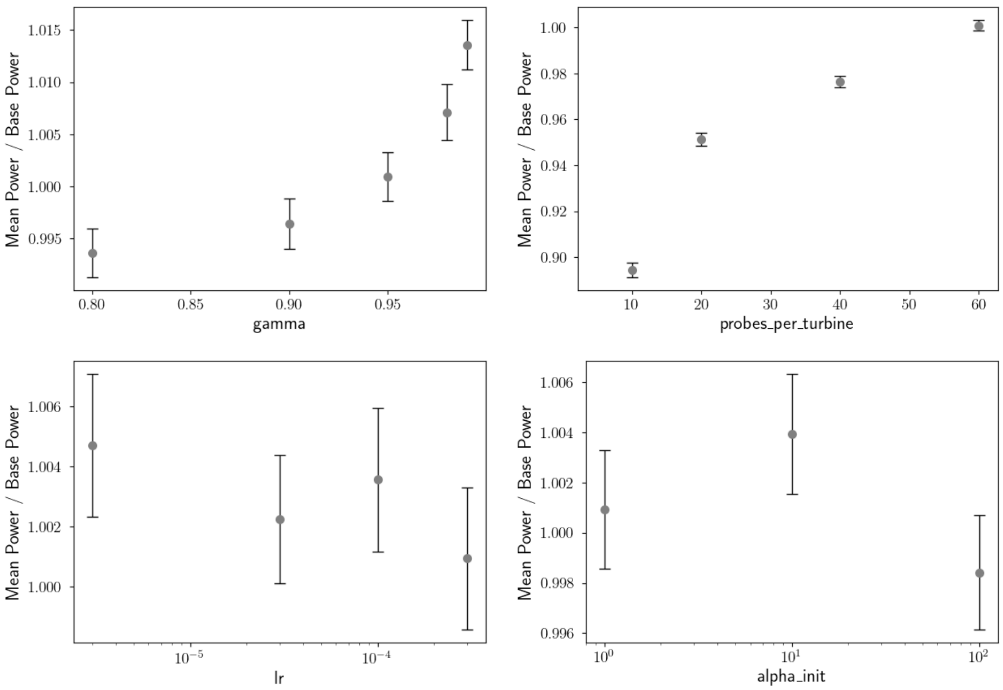

Figure S4: Parameter sweep results. The impact on wind farm power output during evaluation of RL controllers trained with varying *(a)* discount factor (γ), *(b)* number of probes per turbine, *(c)* learning rate for both the actor and critic networks, and *(d)* initial entropy loss multiplier. Each panel presents the results of sweeping one parameter while holding others constant, with performance evaluated based on the rewards obtained from an evaluation of the trained controller. Error bars show the 95% CI computed over $n = 224$ evaluation environments.
